# Combined *in silico* and ^19^F NMR analysis of 5-fluorouracil metabolism in yeast at low ATP conditions

**DOI:** 10.1042/BSR20192847

**Published:** 2019-12-10

**Authors:** Piotr H. Pawłowski, Paweł Szczęsny, Bożenna Rempoła, Anna Poznańska, Jarosław Poznański

**Affiliations:** 1 Institute of Biochemistry and Biophysics, Polish Academy of Sciences, Warsaw, Poland; 2 Institute of Experimental Plant Biology and Biotechnology, Faculty of Biology, University of Warsaw, Warsaw, Poland; 3 National Institute of Public Health-National Institute of Hygiene, Department of Population Health Monitoring and Analysis, Warsaw, Poland

**Keywords:** 5 fluorouracil, anticancer drug, metabolic pathways

## Abstract

The cytotoxic effect of 5-fluorouracil (5-FU) on yeast cells is thought to be mainly via a misincorporation of fluoropyrimidines into both RNA and DNA, not only DNA damage via inhibition of thymidylate synthase (TYMS) by fluorodeoxyuridine monophosphate (FdUMP). However, some studies on *Saccharomyces cerevisiae* show a drastic decrease in ATP concentration under oxidative stress, together with a decrease in concentration of other tri- and diphosphates. This raises a question if hydrolysis of 5-fluoro-2-deoxyuridine diphosphate (FdUDP) under oxidative stress could not lead to the presence of FdUMP and the activation of so-called ‘thymine-less death’ route. We attempted to answer this question with *in silico* modeling of 5-FU metabolic pathways, based on new experimental results, where the stages of intracellular metabolism of 5-FU in *Saccharomyces cerevisiae* were tracked by a combination of ^19^F and ^31^P NMR spectroscopic study. We have identified 5-FU, its nucleosides and nucleotides, and subsequent di- and/or triphosphates. Additionally, another wide ^19^F signal, assigned to fluorinated unstructured short RNA, has been also identified in the spectra. The concentration of individual metabolites was found to vary substantially within hours, however, the initial steady-state was preserved only for an hour, until the ATP concentration dropped by a half, which was monitored independently via ^31^P NMR spectra. After that, the catabolic process leading from triphosphates through monophosphates and nucleosides back to 5-FU was observed. These results imply careful design and interpretation of studies in 5-FU metabolism in yeast.

## Introduction

**Figure 1:**
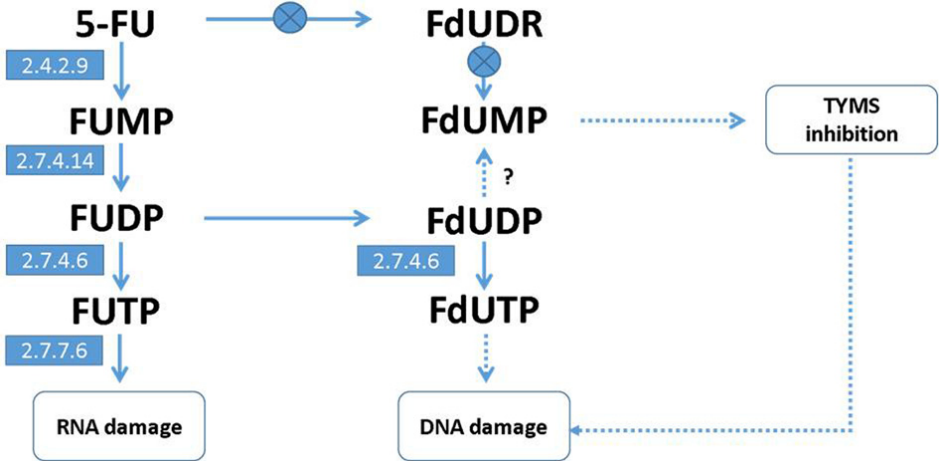
The overview of 5-FU metabolism in yeast The absence of TYMP and TK1 that are present in higher organisms is indicated by crossed circles. FdUMP, which inhibits thymidylate synthase (TYMS) causes imbalance of deoxynucleotide pool. Triphosphates, FUTP and FdUTP, may be further incorporated into RNA and DNA, respectively. The reactions leading to these triphosphates are stemming from a common intermediate, FUMP, while FdUMP is not a product of an alternative route, through FUDR, catalyzed by TYMP and TK1, as in human.

Modeling and analysis of metabolic pathways have received an increasing amount of attention over the past two decades [1–3]. This includes the analysis of the consequences of change in enzyme expression and activity profiles, and further, predicting modification of the key features of metabolism, including growth yield, metabolite distribution or system robustness. Moreover, this approach allows identification of gene control by metabolites and the associated enzymes [4]. To achieve these goals successfully, a reconstruction draft of the metabolic network topology and the detailed mathematical models of metabolic processes, breaking into reactions and enzymes, are required. Despite the fact that the kinetics of chemical reactions is the most frequently tested, evaluated and debugged observable, there is still inadequate knowledge of the enzyme–kinetic rate laws [5,6], in particular of values associated with microscopic rates [7]. In the following paper, we present how we dealt with this encumbrance by multi-species fitting of the general kinetic model to the entire set of consistent time course experimental data, regarding the 5-fluorouracil (5-FU) post-incubation intracellular metabolism. 5-FU is one of the most widely used anticancer drugs [8], whose cytotoxicity is associated with its metabolites. These are the fluorodeoxyuridine monophosphate (FdUMP), which inhibits thymidylate synthase causing deoxynucleotide pool imbalance and two triphosphates, the fluorouridine triphosphate (FUTP) and the fluorodeoxyuridine triphosphate (FdUTP), which are misincorporated into RNA and DNA, respectively (reviewed in [9]). The reactions leading to these triphosphates are stemming from a common intermediate, the 5-fluorouridine monophosphate (FUMP), while FdUMP is mainly a product of an alternative route, through fluorodeoxyuridine (FUDR), catalyzed by thymidine phosphorylase (TYMP) and thymidine kinase (TK1). See Figure 1 for a schematic overview of 5-FU metabolism in yeast.

*Saccharomyces cerevisiae* is a convenient and inexpensive model to test the response of anticancer compounds [10], due to its structure of many cellular processes homologous to humans. However, in case of 5-FU, its metabolic capabilities differ substantially. Wild-type yeast have neither the thymidine phosphorylase [11,12] nor the thymidine kinase [13], therefore they are incapable to effectively produce FdUMP. As such, the major cytotoxic effect of 5-FU in yeast is due to misincorporation of fluoropyrimidines into ribonucleic acids [14,15]. This creates a unique opportunity to study independently the cellular effects of 5-FU and FdUMP [16]. Dephosphorylation of the 5-fluoro-2-deoxyuridine diphosphate (FdUDP) to the monophosphate (FdUMP) has been postulated by Longley and co-workers [9] in human cells, but no evidence of such process in yeast has been found so far.

Bakers’ yeast can metabolize glucose using the fermentative or oxidative pathway, depending on the concentration of glucose [17]. Both conditions differ by the response of the cells to oxidative stress. The addition of H_2_O_2_ (0.05–1.0 mM) to the yeast cultures with low (0.025%) and high (2%) glucose concentration resulted in a sharp decrease in ATP and a less extensive decrease in GTP, CTP, UTP and ADP levels, respectively, [18,19] in fermentative conditions. According to our best knowledge, this effect in the context of 5-FU metabolism was studied only by Sillero and co-workers [20], who looked at levels of 5-fluoro-UDP-sugars in fermentative and oxidative (high and low concentrations of glucose, respectively) conditions.

A decrease in triphosphates in response to oxidative stress raises a question if the shift toward catabolic processes caused by absence of ATP could be the source of FdUMP in 5-FU metabolism in yeast. Here, we simultaneously followed levels of 5-FU metabolites and ATP in the *S. cerevisiae* cytoplasm using NMR spectroscopy. We observed the increase in FdUMP concentration already during the drop in ATP concentration, and even sharper increase in FdUMP once ATP has been completely depleted.

## Materials and methods

### Biological sample

The strain used in this work was the wild-type *S. cerevisiae* W303, (*MAT*a/*MAT*α *ura3-1/ura3-1, leu2-3/leu2-3, his3-11/his3-11, trp1-1/trp1-1, ade2-1/ade2-1, can1-100/can1-100)*, [21] transformed with pRS426 plasmid [22]. Yeast cells were grown at 28°C in synthetic complete (CSM-drop-out) medium lacking uracil with 2% glucose as a carbon source, prepared according to the manufacturer’s instructions (Bio 101).

The growth rate and survival of yeast cells in the presence of 0.0, 0.1, 0.2, 0.4 and 0.8 mM 5-FU was measured to determine the optimal 5-FU concentration in the growth medium, which slightly affected the growth rate and allowed to follow its concentration in the cells. Cells growth in the presence of 5-FU was followed by measurement of optical density at λ = 600 nm. Cell viability was assessed after 4-h incubation with 5-FU (0–10 mM) by plating serial dilutions of the yeast cultures on YPD plates (1% yeast extract, 2% peptone, 2% glucose, 2% agar), and counting the colonies formed after incubation at 28°C for 2 days.

To prepare samples for NMR measurements, exponentially growing yeast cells (1 L) were treated with 0.1 mM 5-FU for 12 h. Yeast cells were then pelleted by centrifugation, washed twice with water, resuspended in 0.2 mM Tris buffer at pH 7.5 (12 ml) and disrupted with glass beads of 0.5 mm diameter) by vortexing at the top speed of a tabletop mixer for 10 periods of 1 min separated by 1-min periods of cooling on ice. Cell debris was removed by three rounds of centrifugation (8000 rpm for 15 min and twice at 14000 rpm for 15 min). Resulting supernatant was divided into 600-µl portions, mixed with 10 µl ml D_2_O and directly subjected to NMR measurement or stored at −80°C. After each centrifugation, including washing of the cells, the samples of supernatant were collected to determine the 5-FU content.

### NMR experiments

All NMR spectra of the samples of yeast cytoplasmic fraction were recorded on Varian Inova-400 MHz spectrometer, and processed with MestreC 4.5 [23]. Seventeen succeeding ^19^F spectra, each acquired for 1 h, were analyzed in the pseudo 2-D mode to minimize possible disruptions. All these spectra were collected at 376.32 MHz on a spectrometer equipped with a 5-mm inverse broadband pfg probe at 25°C using an s2pul pulse sequence with a spectral width of 8.0 kHz, 16000 data points and 488 scans. The recycle time of 7.4 s (i.e. relaxation delay of 5.4 s) was used as a compromise between the standard qNMR approach [24] and maximum S/N ratio achievable in 1-h experiment. π/2 shifted square-sine bell and Lorenz filter (LB = 0.5Hz) followed by zero-filling to 16k was applied prior to Fourier transformation. The signal of FU signal (approximately −172.1 ppm) was used as the internal reference.

To monitor the balance of ATP and its metabolites, a series of ^31^P spectra was additionally recorded at 1-h intervals for a part of the same sample, which was temporarily stored at −80°C to minimize uncontrolled spontaneous catabolic reactions. ^31^P NMR spectra of cytoplasm samples were collected at 161.897 MHz. Fourteen spectra were acquired in 1-h intervals at 25°C using an s2pul pulse sequence with a spectral width of 6.5 kHz, 20802 points and 384 scans. Since only the time evolution of a particular resonance was monitored, the repetition time was reduced to 10 s to enhance S/N ratio. Additional inspection of the intensity of H1′ and H8 resonances of ATP/ADP (6.17/8.47 ppm) and GTP/GDP (5.93/8.13 ppm) [25,26] in the spectrum recorded for the same cytoplasm sample (not shown) enabled raw estimation of the relative concentration of GTP+GDP vs. ATP+ADP to 1:5, which agrees with the literature data [27].

### The kinetic model of 5-FU metabolism

Twenty-two reactions and sixteen species for pyrimidine metabolism in *S. cerevisiae* (budding yeast), listed in Table 1, were taken from the Kegg Pathway Database [28] to analyze the *in silico* model of 5-FU metabolism in yeast.

**Table 1:** The kinetic model of 5-FU metabolism (according to Kegg database)

Number	Name	Reaction	Rate Law
1.	2.4.2.9 uracil phosphoribosyltransferase	FUMP = 5FU	Rev. Michaelis–Menten
2.	2.7.4.14 UMP/CMP kinase	ATP + FUMP = ADP + FUDP	Ping Pong Bi Bi
3.	2.7.4.6 (1) nucleoside-diphosphate kinase	ATP + FUDP = ADP + FUTP	Ping Pong Bi Bi
4.	2.7.7.6 DNA-directed RNA polymerase	FUTP → FRNA	Irr. Michaelis–Menten
5	3.6.1.5 (1) UTP phosphohydrolase	FUTP → FUDP	Irr. Michaelis–Menten
6.	3.6.1.5 (2) UDP phosphohydrolase	FUDP → FUMP	Irr. Michaelis–Menten
7.	*3.1.3.5 (*1*) hipotetical*	FUMP → FUDR	Irr. Michaelis–Menten
8.	2.7.1.48 uridine kinase	ATP + FUDR = ADP + FUMP	Ping Pong Bi Bi
9.	3.2.2.3 uridine hydrolase	FUDR → 5FU	Irr. Michaelis–Menten
10.	1.17.4.1 UDP reductase	FUDP → FdUDP	Irr. Michaelis–Menten
11.	2.7.4.6 (2) nucleoside-diphosphate kinase	ATP + FdUDP = ADP + FdUTP	Ping Pong Bi Bi
12.	3.6.1.23 dUTP nucleotidohydrolase	FdUTP → FdUMP	Irr. Michaelis–Menten
13.	2.7.4.9 (1) dUMP kinase	ATP + FdUMP = ADP + FdUDP	Ping Pong Bi Bi
14.	2.1.1.45 thymidylate synthase	FdUMP → FdTMP	Irr. Michaelis–Menten
15.	*3.1.3.5 (*2*) hipotetical*	FdUMP → FdUDR	Irr. Michaelis–Menten
16.	2.4.2.1 purine-nucleoside phosphorylase	FdUDR → 5FU	Irr. Michaelis–Menten
17.	*2.7.7.8 - hipotetical*	FRNA = FUDP	Rev. Michaelis–Menten
18.	2.7.4.6 (3) nucleoside-diphosphate kinase	ATP + FdTDP = ADP + FdTTP	Ping Pong Bi Bi
19.	2.7.7.7 DNA-directed DNA polymerase	FdTTP → FDNA	Irr. Michaelis–Menten
20.	2.7.4.9 (2) dTMP kinase	ATP + FdTMP = ADP + FdTDP	Ping Pong Bi Bi
21.	3.6.1.5 (3) dTTP phosphohydrolase	FdTTP → FdTDP	Irr. Michaelis–Menten
22.	3.6.1.5 (4) dTDP phosphohydrolase	FdTDP → FdTMP	Irr. Michaelis–Menten

**Figure 2:**
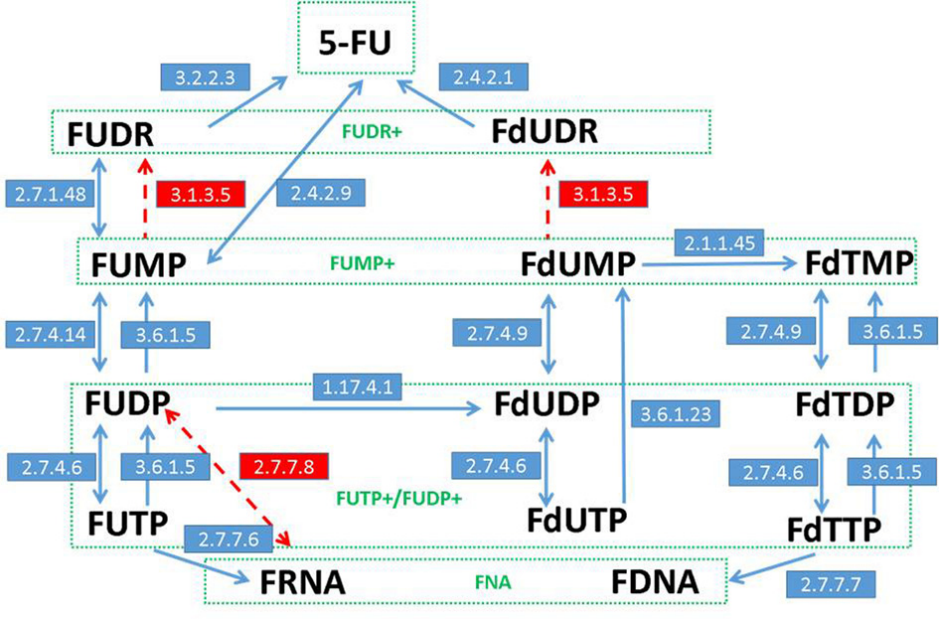
The general schema of the proposed kinetics model of 5-FU metabolism Reaction descriptions are presented in Table 1. Red color indicates not-confirmed experimentally hypothetical enzymes and pathways, introduced to provide a functionality of absence in the yeast EC 3.1.3.5 and EC 2.7.7.8. Green boxes indicate species, the total concentration of which was determined experimentally.

The presumed physiological directions of the investigated metabolic reactions have been chosen according to the Kegg pathway maps (sce00240) indications. Chosen species and moieties (Figure 2 in green rectangles) obey fluorinated analogs: of uracil (5-FU), of uridine and deoxyuridine (FUDR+), of derivative monophosphates (FUMP+), and of derivative diphosphates/triphosphates (FUTP+/FUDP+), which were analyzed as a whole (total concentration). Red marked enzymes have not been reported by Kegg as natural for *S. cerevisiae*, but were used in the proposed model as the hypothetical analogs that mimic return pathways.

### Numerical fitting of the model

The kinetic model of 5-FU metabolism was fitted to the experimental data representing the evolution of 5-FU metabolites and ATP concentrations over time. All the data for 5-FU metabolites recorded during the period 1–17 h were used in the simulations. The time-evolution of particular metabolite concentration was fitted individually, and then summed for groups of compounds indistinguishable in ^19^F NMR spectrum (e.g. 5-FUrd and 5-FdUrd). The experimental ^31^P NMR data collected in the period 1–14 h were extrapolated to the range of 0–17 h using the scale-free approximation with the relative ATP level (t = 0) set to 1.

In general, the properties of the same enzyme acting on different species were distinguished and the specific attributes marked by the successive natural numbers (i = 1, 2…), e.g. 2.7.4.9 (1) and 2.7.4.9 (2), for dUMP and dTMP kinase.

To avoid the fitting ambiguity, the well-documented value of Michaelis constant, *K*_m_, for the phosphohydrolase 3.6.1.5 (1)-(4) was fixed (according to Brenda database) to *K*_m_ = 12.5 µM (for UDP, dTDP) and *K*_m_ = 15 µM (for UTP, dTTP). However, to seriously reduce the number of investigated items and this way the error of the estimation, the parameters of the other enzymes, acting for different substrates, were treated as invariant. Analyzed reactions are listed in Table 1.

The Copasi 4.22 simulator for biochemical networks [29] were used to estimate 113 parameters (initial concentrations and the rate law parameters) in the considered 5-FU metabolism model (Table 1). The applied rate law formula: irreversible and reversible Michaelis–Menten, and Ping Pong Bi Bi are cited below:


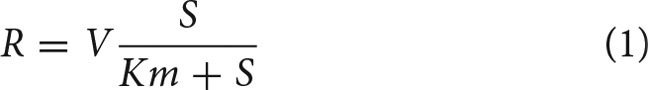



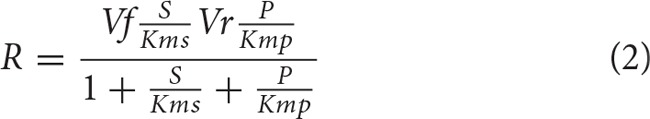



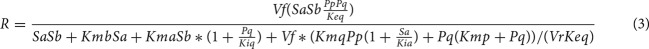


where symbols meant: V, maximum rate (f/r, forward/reverse); *K*_m_, apparent Michaelis conts. (S/P, substrate/product); *K*_i_, inhibition const., *K*_eq_, equilibrum constant.

After the preliminary tests with different parameter estimation methods, start values and the bounds, the Hooke and Jeeves heuristic method (it. lim. 50. tol. 1e-5. Rho 0.2, scaled standard deviation weights), and the respectively wide enough parameter ranges, were chosen to pre-minimize the objective function (weighted least squares) below arbitrary value 0.15, to ensure the freedom, and the initial precision, in the following parameter search. Then, several consecutive attempts using the same method and the previous adjustments as starting points were performed till the variation in the value of the objective function was less than 10^−6^. Finally, only the five most accurate attempts were considered. For each investigated parameter, the best fit value (bfv), the average value of analyzing attempts (avg), the standard deviation of the sample (stds) and the dev/stds ratio (where dev is the absolute value of the deviation of the bfv from the average value) were analyzed.

The question how much a change in a concentration of a given substrate can modify the reaction rate of an isolated enzyme, or how much the change in an activity of a given enzyme can modify a given pathway flux or a concentration of a metabolite was resolved by the metabolism control analysis (MCA).

## Results

### Estimation of EC_50_ for 5-FU

The effect of 5-FU concentration on cell growth was monitored by means of the optical density at λ = 600 nm (OD in Table 2). The inhibition of cell growth was observed for all tested concentrations of 5-FU (Supplementary Figure S1).

**Table 2:** Optical density of growing cell cultures

[FU]/mM	OD (t = 0 h)	OD (t = 4 h)	OD (t = 18 h)
0.8	0.22	0.43	0.34
0.4	0.22	0.47	0.36
0.2	0.22	0.40	0.37
0.1	0.22	0.39	0.32
0	0.22	1.00	2.96

**Figure 3:**
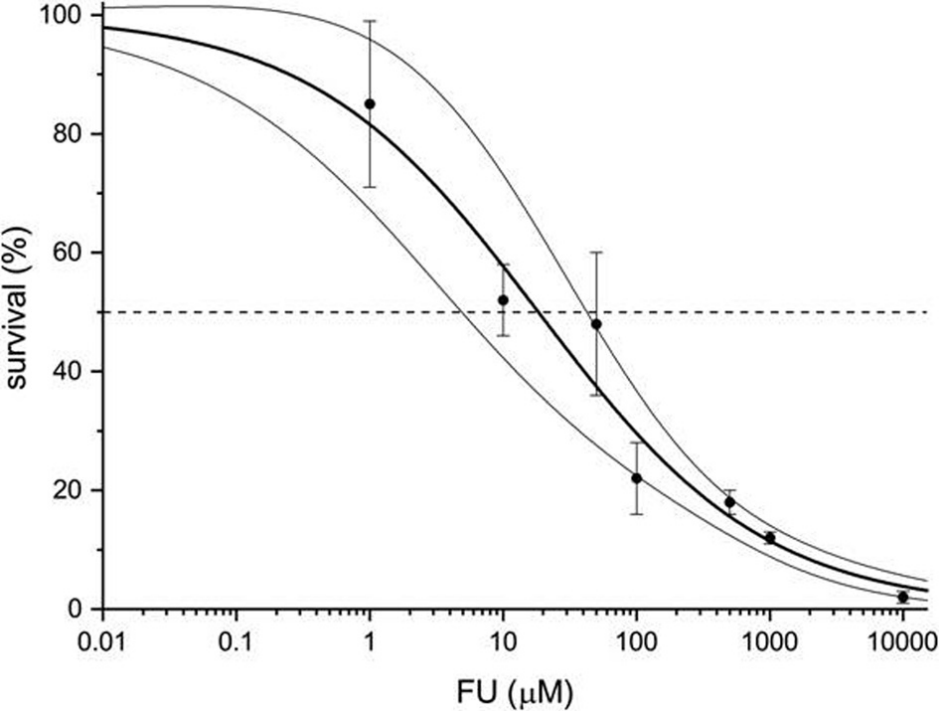
The log plot of dose–survival relation determined for *S. cerevisiae* W303 strain Black circles represent the mean of five independent repetitions, while thin vertical lines denote associated variation. The thick line represents the best fit of the formula: survival = 100%/(1+ (FU/EC_50_)^−Hill)^, while the thin lines boarder 95% confidence band.

The dose–survival curve (Figure 3) clearly demonstrated that the studied W303 strain of *S. cerevisiae* is highly susceptible to the 5-FU treatment (EC_50_ = 18 ± 7 µM, Hill = 0.51 ± 0.06), the concentration of which was approximately 1 mM seemed to guarantee penetration of 5-FU into the cell interior and the relative robustness of the cellular metabolism. Consequently, 1 mM 5-FU was used in all further experiments.

### Evidence of 5-FU penetrating yeast cell interior

**Figure 4:**
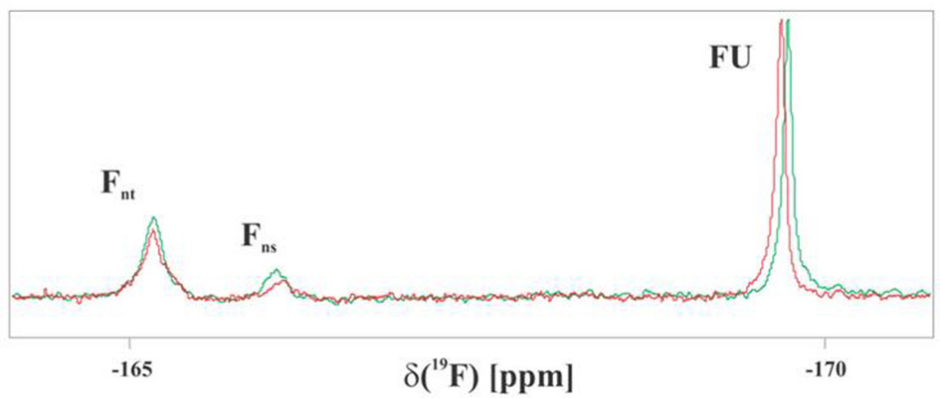
^19^F NMR spectra acquired for the yeast cells packed in the 5-mm NMR tube The spectra were recorded 1 (green) and 11 (red) h after deposition of washed cells, previously pre-treated for 1 h by 1 mM 5-FU in the feeding medium. FU, F_ns_ and F_nt_ denote resonances attributed to 5-FU, its nucleosides and their 5′-phosphates, respectively.

Figure 4 presents ^19^F NMR spectra obtained for yeast after a short (1-h) incubation with low concentration (1 mM) of 5-FU. The spectra recorded immediately and 11 h (marked in red) after the incubation, both demonstrate that 5-FU efficiently penetrates the yeast cell interior. Contrary to the Parisot study on *Nectria haematococca* [30], beside 5-FU and its nucleotides we also observed fluorinated nucleosides, but not α-fluoro-β-alanine (FBAL). Small, but distinct differences in the relative intensities of resonances are attributed to nucleosides and their 5′-phosphates and indicate the metabolism of 5-FU by the yeast cells. The shift of FU resonance location is indicative of the small change in intracellular pH.

It should be noted that extreme resonance broadening that results from inhomogeneous and variable distribution of cells spontaneously sedimenting in the NMR tube precluded any quantitative analysis of the data. This could be, however, overcome by either immobilization of cells or sole analysis of extracted cytoplasmic fraction.

### Time evolution of ATP concentration

Relative changes in the ATP concentration were analyzed in ^31^P NMR spectra recorded in pseudo-2D model (not shown). The concentration of ATP drops within 4 h, nearly to zero (see numerical fitting of the model). During the next 13 h, we did not observe any process leading to the production of ATP.

### Time evolution of 5-FU metabolites

^19^F NMR spectra, 1 h each, were recorded successively for 17 h.

**Figure 5:**
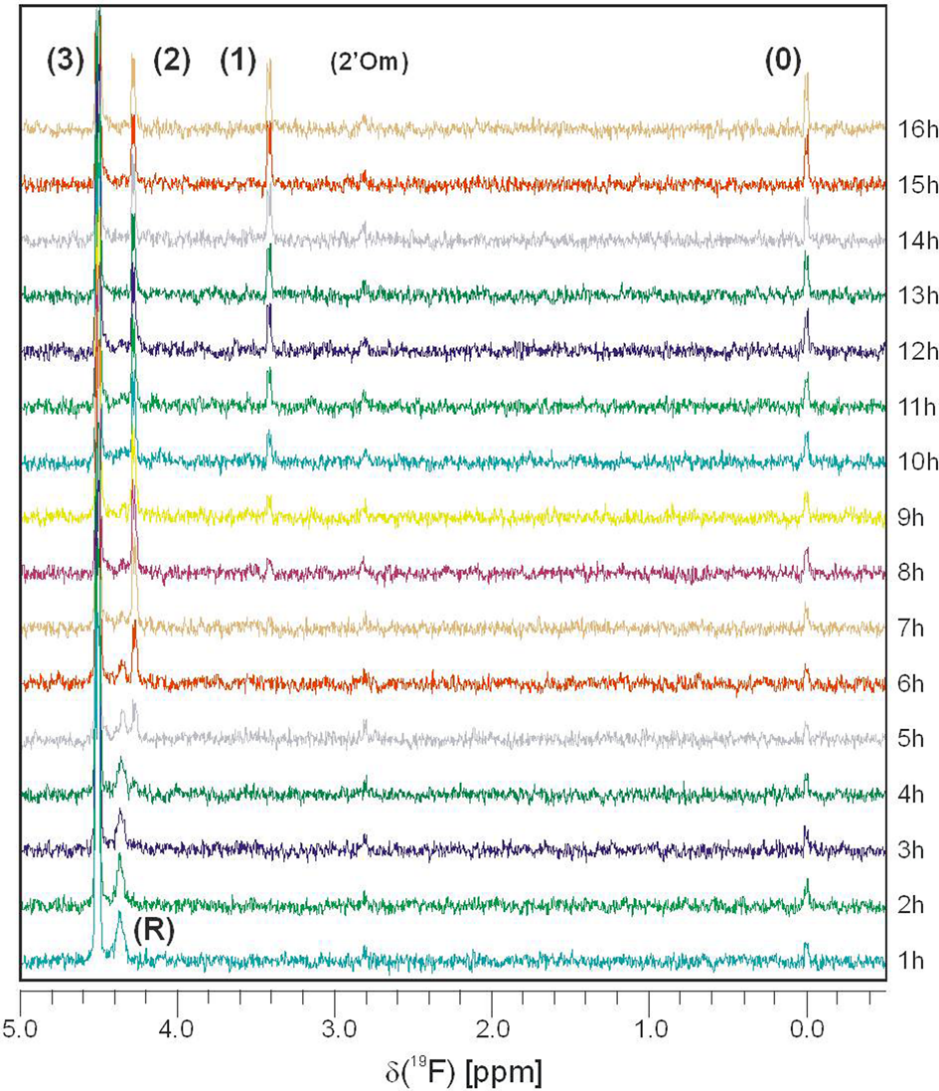
Time evolution of ^19^F NMR spectrum The evolution is shown as a superposition of 16 spectra, recorded for cytoplasmic fraction of yeast cells, which were grown in the medium with 0.1 mM 5-FU. In these spectra, which were recorded in 60-min intervals, resonance lines attributed to 5-FU (0), its nucleosides (1), corresponding to mono- and di- (2) and triphosphates (3) were unequivocally assigned. The location of the wide resonance (R) (4.4 ppm upfield to FU) is indicative for FU in short unstructured RNA (20–30 nt). Assignment of the minor signal (2′Om) to 2′-*O*-methyl-5-fluorouridine has been supported by inspection of MODOMICS database [40] against the spectral data for FU derivatives [41].

In all recorded ^19^F NMR spectra 5-FU, its nucleosides, and corresponding mono-, di- and triphosphates were assigned (Figure 5). Both ribo- and deoxyribonucleosides are assigned together in the spectra due to their signals’ overlap. The halfwidth of the broad signal located downfield to that of FdUMP/FUMP (∼17 Hz) corresponds to the transverse relaxation time (T2) of 20 ms, when the Spin-Spin Relaxation regime is assumed [31]. Consequently, T2 of 20 ms implies that the rotational correlation time (τ_c_) fails in the range of 1–10 ns, the value of which is characteristic for small RNA (20–30 nt), while for tRNA τ_c_ was estimated to be ∼23 ns [32]. Location of the signal (4.4 ppm upfield to 5-FU) agrees with ^19^F spectrum recorded of poly(FU) (^19^F NMR studies of the solution structure and dynamics of 5-FU-substituted valine tRNA from *Escherichia coli* [33,34]), which is unstructured in solution [35] or 5-FU in the unstructured regions of tRNA [36], which is efficiently incorporated [37]. Summarizing, the wide signal (R in Figure 5) must be assigned to 5-FU in short unstructured RNA fragments (20–30 nt), while extremely slow tumbling precludes direct recording of ^19^F incorporated either DNA or mRNA/rRNA. These short oligo-RNA do exist in yeast cells (miRNAs, piRNA, qiRNA, siRNA, or tRFs) [38,39], but may also originate from sonication-induced RNA fragmentation (shearing). The population of unstructured short RNA decreases rapidly, and no such molecules are observed after 8 h.

We also observe weak signal (marked in Figure 5 as 2′Om) from another fluorinated low-mass compound. Inspection of all known uridine modifications listed by the MODOMICS server [31,42] pointed 2′-O-methyl-5-FUrd as the only metabolite that likely fits the observed signal location in the ^19^F spectrum [32]. The location is also close (but not identical) to the value reported for 5-FC [43], however this alternative assignment is less probable since no back-conversion of 5-FU into 5-FC has been reported yet. As time progresses, we observe a small but steady increase in its concentration.

**Figure 6:**
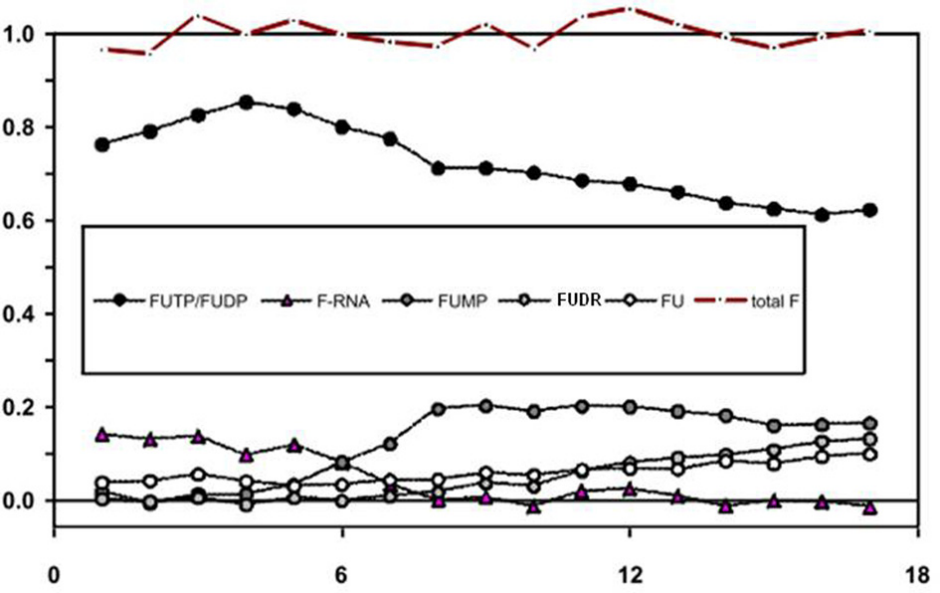
Time evolution of the relative concentration of particular fluorinated species For each compound, its concentration was estimated by integration of the appropriate signal assigned in the ^19^F NMR spectrum.

As the time evolution of signal intensities represents the changes in concentration, we have normalized the area under the curves of all assigned compounds to the initial concentration of 5-FU being 1 mM. Figure 6 shows the time course of all assigned compounds together with the sum of their concentrations (total F).

In Figure 6, we observed an increase in concentrations of FUTP/FUDP (including their deoxy forms) during the first 4 h of the measurements, which corresponds to the presence of ATP in the sample. Also, during that time, we detect a steady signal from 5-FU incorporated into RNA. Then, the ATP signal disappears and the signals indicating concentrations of FUTP/FUDP and 5-FU incorporated into RNA start to decrease, while the signal corresponding to the concentration of FUMP/FdUMP increases. In case of free 5-FU and FdUDR, their time evolution of the concentration does not have any dramatic turn of events—both have a larger concentration after 17 h at the end of the experiment than at the beginning. The total concentration of all fluorinated compounds remained constant during the experiment, indicating that the majority of flurorinated compounds have been identified, and no additional reactions beyond the ones assigned/defined in the initial model (Figure 1) significantly contribute to 5-FU metabolism.

## Numerical results

**Figure 7:**
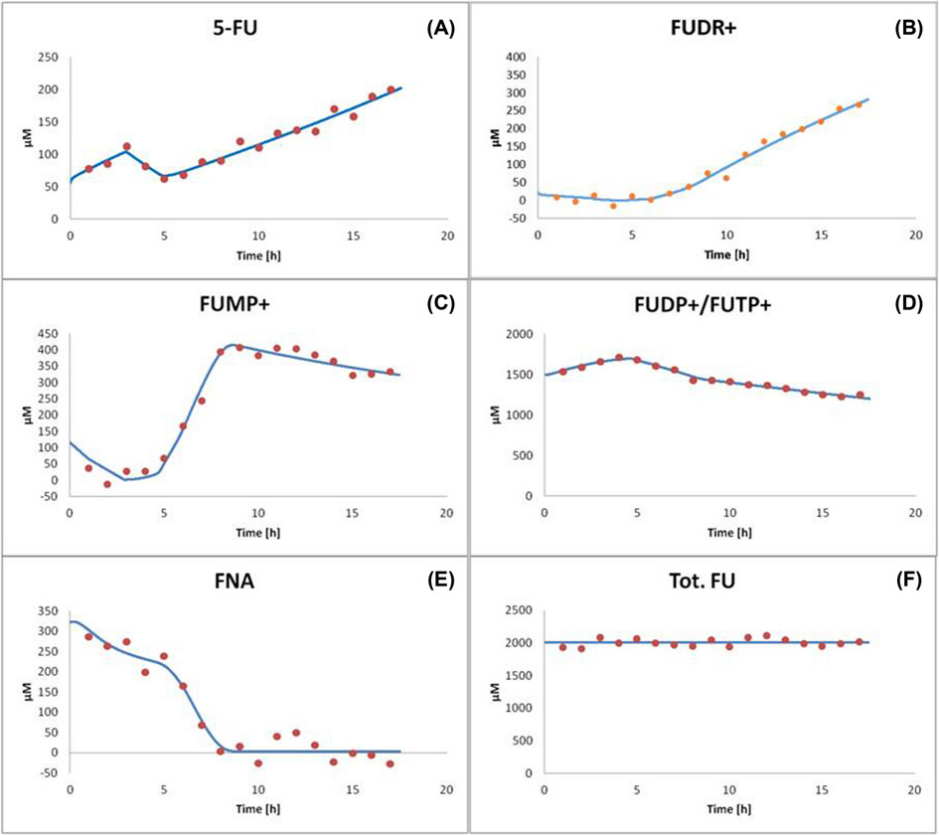
The best fit obtained for the best model of 5-FU metabolism The estimated values of parameters are summarized in Tables 3 and 4. Particular panels show (**A**) the time evolution of 5-FU. (**B**) The time evolution of FUDR+. (**C**) The time evolution of FUMP+. (**D**) The time evolution of FUDP+/FUTP+. (**E**) The time evolution of FNA. (**F**) The time evolution of tot. FU. Markers: bold dots, experiment; line, best modeling fit.

**Figure 8:**
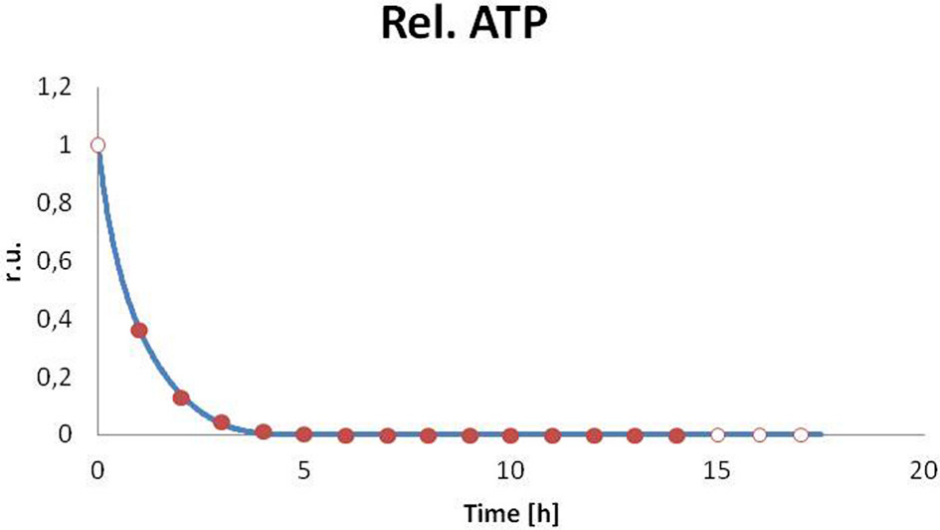
The best fit of the ATP catabolism model The evolution of ATP (relative value): solid and empty circles show the data extracted from ^31^P NMR spectra and extrapolation, respectively; blue line represents numerical fitting.

The bfv, avg, stds, and the dev/stds ratio were analyzed in the Tables 3 and 4. The plots reflecting the best fit were shown in Figures 7A–F and 8.

**Table 3:** Kinetic parameters [µM].[µM/h]

R. no.	Enzyme	Parameter	Bfv	Avg	Stds	Dev/Stds
1.	2.4.2.9	Kmp	3.3443E+01	3.3460E+01	2.2E-02	0.79
		Kms	2.5724E+03	2.5702E+03	2.4E+00	0.94
		Vf	3.225E+01	3.204E+01	2.0E-01	1.08
		Vr	2.82188E+01	2.8173E+01	3.4E-03	0.45
2.	2.7.4.14	Keq	4.3235E+07	4.3171E+07	7.5E+04	0.85
		Kia	6.882E-13	6.881E-13	1.0E-15	0.12
		Kiq	7.7E+07	7.9E+07	1.1E+07	0.17
		Kma	3.527E-12	3.560E-12	2.8E-14	1.18
		Kmb	2.29E-08	2.25E-08	1.2E-09	0.35
		Kmp	1.20E-02	1.05E-02	1.8E-03	0.85
		Kmq	8.487E-17	8.465E-17	3.0E-19	0.74
		Vf	1.8201E+02	1.8214E+02	1.2E-01	1.15
		Vr	2.6238E-01	2.6234E-01	1.2E-04	0.32
3.	2.7.4.6 (1)	Keq	4.6890E+00	4.6875E+00	2.5E-03	0.59
		Kia	2.8129E+05	2.8252E+05	8.5E+02	1.45
		Kiq	8.9666E-01	8.9635E-01	8.6E-04	0.37
		Kma	4.141E+00	4.131E+00	1.3E-02	0.82
		Kmb	3.7026E-04	3.7097E-04	9.7E-07	0.73
		Kmp	2.18E-03	2.13E-03	1.1E-04	0.45
		Kmq	6.214E+00	6.224E+00	2.5E-02	0.42
		Vf	3.1626E+03	3.1594E+03	3.9E+00	0.81
		Vr	1.7793E+02	1.7758E+02	5.0E-01	0.71
4.	2.7.7.6	Km	3.576E+04	3.584E+04	1.3E+02	0.65
		V	7.198E+02	7.184E+02	2.0E+00	0.71
5.	3.6.1.5 (1)	Km	=1.500E+01	=1.500E+01	0	0
		V	1.45717E+02	1.45722E+02	1.2E-02	0.45
6.	3.6.1.5 (2)	Km	=1.250E+01	=1.250E+01	0	0
		V	As for R.no.5	As for R.no.5	As for R.no.5	As for R.no.5
7.	3.1.3.5 (1)	Km	4.07511E-06	4.07548E-06	8.2E-10	0.45
		V	3.41039E+01	3.40999E+01	6.3E-03	0.64
8.	2.7.1.48	Keq	5.015E+11	4.968E+11	4.4E+09	1.09
		Kia	2.2328E+05	2.2335E+05	4.9E+02	0.13
		Kiq	2.6E+07	2.6E+07	1.3E+07	0.01
		Kma	1.6790E-04	1.6825E-04	4.9E-07	0.72
		Kmb	7.9E-08	7.3E-08	1.4E-08	0.45
		Kmp	1.66E-04	1.82E-04	1.8E-05	0.92
		Kmq	1.9503E+04	1.9415E+04	9.3E+01	0.95
		Vf	3.73846E+01	3.73837E+01	3.6E-03	0.27
		Vr	4.8109E-01	4.8052E-01	8.2E-04	0.70
9.	3.2.2.3	Km	1.04249E+05	1.04249E+05	4.8E+01	0.01
		V	3.06650E+03	3.06640E+03	1.0E-01	1.00
10.	1.17.4.1	Km	7.28274E+03	7.28224E+03	6.5E-01	0.78
		V	3.888900E+03	3.888910E+03	6.6E-02	0.15
11.	2.7.4.6 (2)	Same as for R. no. 3				
12.	3.6.1.23	Km	2.778E+05	2.783E+05	2.5E+03	0.22
		V	3.591E-05	3.578E-05	4.0E-07	0.33
13.	2.7.4.9 (1)	Keq	1.14534E+09	1.14571E+09	8.3E+05	0.45
		Kia			8.9E-02	0.25
		Kiq	1.3801E+01	1.3779E+01	3.0E+02	1.29
		Kma	2.113E+04	2.152E+04	3.1E-04	0.87
		Kmb	5.8383E-01	5.8357E-01	4.5E-07	0.22
		Kmp	1.6393E-04	1.6383E-04	2.7E-08	0.74
		Kmq	8.0E-08	8.9E-08	1.2E-04	0.37
		Vf	3.76E-03	3.72E-03	2.6E-05	0.53
		Vr	2.54472E-01	2.54485E-01	3.3E+04	1.71
			9.4E+04	3.9E+04		
14.	2.1.1.45	Km	3.2476E-03	3.2468E-03	2.5E-06	0.33
		V	3.1E-07	6.2E-07	1.9E-07	1.67
15.	3.1.3.5 (2)	Same as for R. no. 7				
16.	2.4.2.1	Km	2.1040E+03	2.1012E+03	3.1E+00	0.94
		V	3.5787E+04	3.5848E+04	7.3E+01	0.84
17.	2.7.7.8	Kmp	3.2001E+00	3.2025E+00	3.4E-03	0.73
		Kms	4.64288E+01	4.64311E+01	1.3E-03	1.79
		Vf			2.2E+00	0.47
		Vr	3.1738E+03	3.1728E+03	1.5E-01	0.70
			8.119E+01	8.129E+01		
18.	2.7.4.6 (3)	Same as for R. no. 3				
19.	2.7.7.7	Km	4.594E+00	4.633E+00	4.6E-02	0.86
		V	4.350E+01	4.382E+01	2.7E-01	1.23
20.	2.7.4.9 (2)	Same as for R. no. 13				
21.	3.6.1.5 (3)	Same as for R. no. 5				
22.	3.6.1.5 (4)	Same as for R. no. 6				

**Table 4:** MCA

Coefficient		Cause	Effect	Value
EC	Max. neg.	FUDP	2.7.4.14	−7.63E+13
	Max. pos.	ATP	2.7.4.14	4.99E+13
FCC	Max. neg.	3.1.3.5 (1)	2.7.4.14	−1
		2.4.2.9	2.7.4.14	−1
	Max. pos.	3.6.1.5 (2)	2.7.4.14	1
		2.7.1.48	2.7.4.14	1
CCC	Max. neg.	3.1.3.5 (1)	FUMP	−7.92E-15
		2.7.4.14	FUMP	−7.92E-15
		2.4.2.9	FUMP	−7.92E-15
	Max. pos.	3.6.1.5 (2)	FUMP	7.92E-15
		2.7.1.48	FUMP	7.92E-15

Abbreviations: CCC, concentration control coefficient; FCC, flux control coefficient.

The quality of the fit was illustrated in Supplementary Figure S2, presenting the linear regression analysis of the trend in the plot of the fitted variables: predicted vs observed values (coefficient of determination R^2^ = 0.999). Theoretical predictions of the best fit model, i.e. the kinetics of changes in flux, and concentration, at the time range, both, the same as for the experiment (Supplementary Figure S3), and in the long time scale (Supplementary Figure S5), were also presented in Supplementary Material.

### Metabolic control analysis

The maximal positive and minimal negative, the values of the elasticity coefficient (EC), flux control coefficient (FCC) and concentration control coefficient (CCC) were presented in Table 4, indicating cause and effect target.

## Discussion

The absence of endogenous fluorinated compounds in living cells serves a chance to follow a metabolic pathway of potential drugs that are fluorinated. In case of 5-FU, its incorporation into tRNA was observed with ^19^F NMR spectroscopy already in the late seventies [44]. Since then a number of studies *in vitro* and *in vivo* attempted to understand the complexity of the metabolism of fluorinated compounds.

In the present study, we attempted to resolve a certain incongruence in the body of knowledge about 5-FU metabolism in yeast. The absence of key enzymes in yeast (sce00240 in [28]) capable of directly producing FdUMP from 5-FU means that thymidylate synthase is not inhibited. As a result, toxic effect for yeast cells treated with 5-FU stems only from misincorporation of FUTP and FdUTP into nucleic acids. Despite that, many authors still write about thymine-less death in the context of 5-FU-treated yeast cells. Are they wrong? Not quite.

Successful fitting of the proposed theoretical model of 5-FU metabolism (Figures 2 and 7, Supplementary Figure S2, and Tables 2 and 4) to the experimental data reveals the dominant role of the flux through the enzymes: 2.7.4.6 (1), 1.17.4.1, 2.7.4.6 (2), 2.7.4.14, 3.6.1.5 (1) and 3.6.1.5 (2) (Supplementary Figure S3A), and indicates the temporal accumulation and preservation of FUTP, FUDP, FdUDP and FUMP (Supplementary Figure S3B). The question may arise, if to compare the direction of the flux through the enzyme 2.7.4.6 (1, 2) above an hour and that suggested by the official Enzyme Commission for this EC number. In this case, our model predicts the reverse flux (Supplementary Figure S3A), even in a case of Vr less than Vf (Table 3). Despite the fact that ADP, UTP, dUTP and dTTP are reported in the Brenda database as possible substrates for nucleoside-diposphate kinase, this result can be at first glance astonishing. Nonetheless, it is comprehensible. The explanation can come from the fast decrease in ATP and FUDP (additionally compounded by the FUDP outflow through 1.17.4.1) in the initial phase of the experiment, both with excess in FUTP and ATP, tilting the chemical balance (*K*_eq_ = 4.689, Table 3). The estimated a relatively high positive flux through 1.17.4.1 indicates the importance of the deoxy-form production, but a very small flux through 2.1.1.45 and 2.7.4.9 (1, 2) (Supplementary Figure S3A) may be a confirmation of the FU-toxic effect. Thus, the reasonable initial amount of FdTTP (Supplementary Table S1) may be the remainder of the pretoxic incubation period.

The MCA (Table 4) shows that the flux through enzymes 2.7.4.14 could be potentially the most affected due to variation in metabolite concentration, or some enzymes activity. Similarly, the concentration of FUMP could be most affected by the variation in enzyme activity. On the other hand, potentially the most influential are variations in the concentration of ATP and FUDP, the flux through 2.4.2.9, 2.7.1.48, 2.7.4.14, 3.6.1.5 (2) and the hypothetical flux through 3.1.3.5 (1). Additionally, the general analysis of sensitivities (not shown), indicates that the apparent Michaelis constant *K*_ma_, for the reaction ATP and FUDR (Table 1, R.8) catalyzed by the enzyme 2.7.1.48, has the most influencing effect on the non-steady state concentrations of species, important in the behavior of the 5-FU metabolic system, at the time exceeding 9 h.

The main phases of the analyzed experiment were predicted by the presented model and summarized in Supplementary Figure S4A–D. Dominant resultant fluxes were indicated. At the beginning of the post-incubation period (Supplementary Figure S4A) there have been seen still effective FRNA and FDNA upload, and the upload of FdUDR hypothetically corresponding to FdUMP. In the next phase of ∼3 h, there was transfer of metabolites from FRNA and FUTP+ level down to FUDP+ level and the balance of fluxes through 2.7.4.14 and 3.6.1.5 (2) (Supplementary Figure S4B). After 6 h, in the ATP-free phase, metabolites started accumulating at the FUMP+, FUDR+ and 5-FU levels (Supplementary Figure S4C). Then in later (after approximately 9 h) phase 5-FU deoxy-form processing by 1.17.4.1 stopped (Supplementary Figure S4D). Discussed model also predicts the long-term behavior of the system with the asymptotic steady state flux around the triangle 2.4.2.9, 3.1.3.5 (2) and 3.2.2.3 (Supplementary Figure S5A and S6) and stable nonze-ro concentration of ADP, 5-FU, FUDR (Supplementary Figure S5B). Other metabolites in the model vanished down to the relatively very small values.

The bfv of the parameters of the model fall in the distance dev < 2 stds from the average for the five most accurate attempts, what indicates the reasonable fitting. In overall, estimation of the initial concentrations of species (Supplementary Table S1) is worse (stds @ 0.8) than the estimation of the other parameters (Table 3, stds @ 0.69). This may be the effect of the lack of the experimental data for the time = 0. It should be also mentioned that the revealed values of standard *K*_m_ (irr. Michaelis–Menten model), for three essential enzymes (1.17.4.1, 2.4.2.1, 2.7.7.7) fall in the range reported by Brenda database, for three other enzymes they are up to two orders of higher magnitude. In a case of 2.1.1.45, *K*_m_ is 2-order lower, and for hypothetical pathways through 3.1.3.5, *K*_m_ is extremely low. It may indicate the toxic effect, the molecular crowding resulting in the difficult accessibility of enzymes and metabolites, or the inadequate modeling requiring further studies. Not enough confirmed activity of 3.1.3.5 in *S. cerevisiae* by leading databases, but demonstrated by a non-zero flux in the performed simulations, may indicate that the functional role of this 5′-nucleotidase could take over 3.1.3.1 and 3.1.3.2. Further, more detailed investigation a kinetic model with the toxic inhibition and a possible replacement taking over the important catalytic function, should be considered.

As Osorio and co-workers had shown [18,19], deficiency of triphosphates can occur in yeast as a result of oxidative stress under fermentative (high glucose) conditions. In the present study, we show that once the ATP is gone from the sample, FUMP/FdUMP appears quickly in the substantial concentration. This shift to catabolic processes is paralleled by the drop to nearly zero of the concentration of 5-FU incorporated into RNA. As there is a growing body of evidence that 5-FU itself induces an oxidative stress [45-47^45–47^], it is likely that under certain conditions, yeast cells might experience the deficiency in triphosphates and as a result, start to produce FdUMP from FdUDP via 2.7.4.9. If true, this could have a substantial impact on studies where the effects of 5-FU and FdUMP were studied separately, like of Matuo and co-workers [16]. Finally, the observed pule of fluorinated RNA may be indicative for the poly(U) polymerase activity, family members of which have been also identified in yeast [48].

Due to differences in the enzymatic repertoire between human and yeast, our results cannot be directly used when studying 5-FU metabolism in human. However, Shin and co-workers [49] had implied low ATP levels as being responsible for 5-FU resistance in colorectal cancer cells. A molecular mechanism behind that observation has not been revealed therefore it is tempting to speculate that ATP plays an important role in 5-FU metabolism in human.

## Supplementary Material

Supplementary Figures S1-S6 and Table S1

## Abbreviations

avg: average value of analyzing attempt
bfv: best fit value
dev/std: dev is the absolute value of the deviation of the bfv from the average value
EC: elasticity coefficient
FdUDP: 5-fluoro-2-deoxyuridine diphosphate
FdUMP: fluorodeoxyuridine monophosphate
FUDR: fluorodeoxyuridine
FUMP: 5-fluorouridine monophosphate
FUTP: fluorouridine triphosphate
MCA: metabolism control analysis
std: standard deviation of the sample
T2: transverse relaxation time
τ_c_: rotational correlation time
5-FU: 5-fluorouracil
