# Supplementary material for: Combined in silico and 19F NMR analysis of 5-fluorouracil metabolism in yeast at low ATP conditions
Source: Biosci Rep. 2019 Dec 10;39(12):BSR20192847. doi: 10.1042/BSR20192847 (PMC6904775; doi:10.1042/BSR20192847)
Supplement: Supplementary Figures S1-S6 and Table S1 [file BSR-2019-2847_supp.pdf]

Supplement

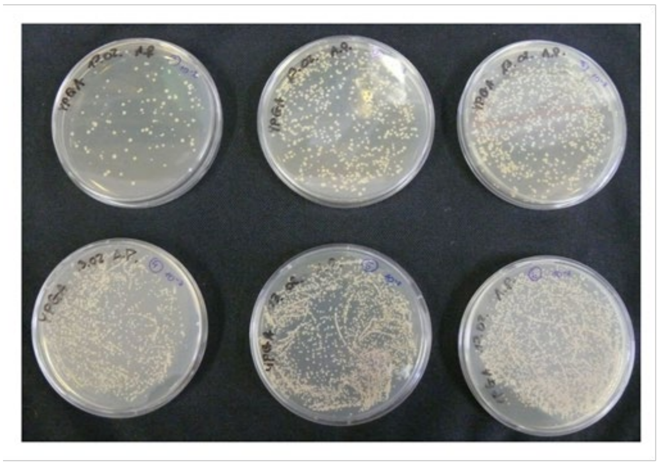

Supp. Fig.1. ***S. cerevisiae*** **growth.** The growth was monitored at 5-FU concentration varying in the range of 10mM - 0.1µM (from top-left to bottom-right, respectively).

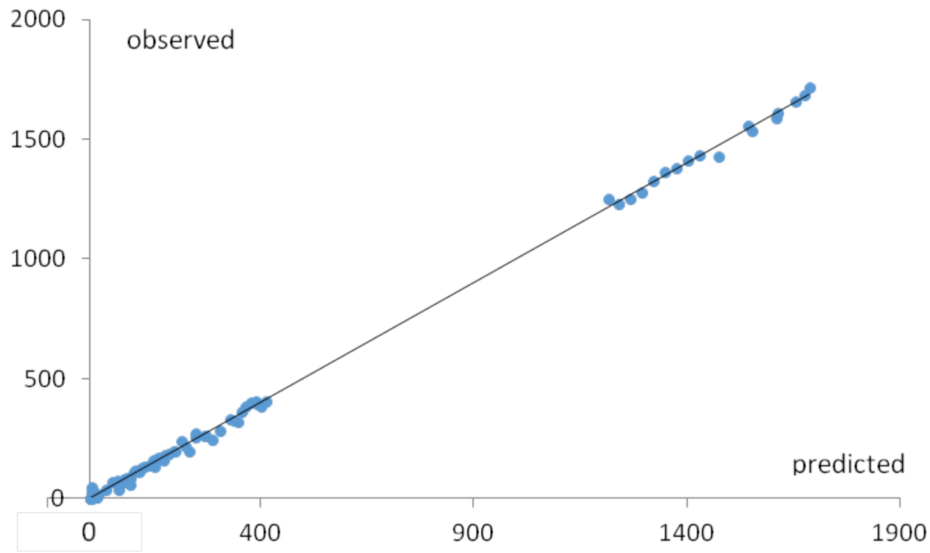

Supp. Fig.2.The quality of fit. Predicted vs observed values, a summary plot for all estimated parameters in an appropriate assumed units. A group of the kinetic rate separates from the concentration related parameters.

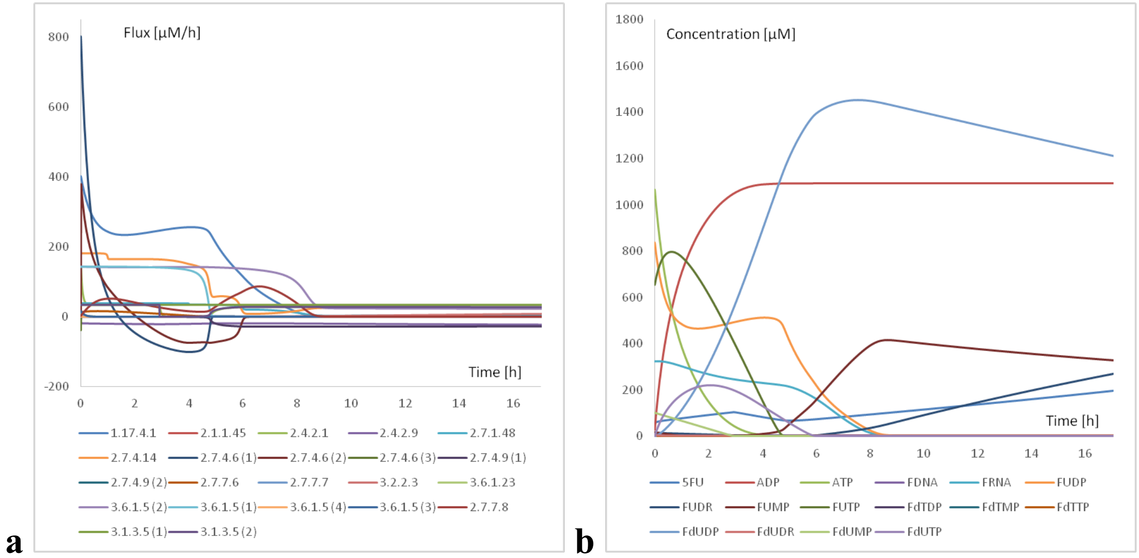

Supp. Fig.3. Theoretical predictions of fitted model (time <=17 h). Kinetics of changes during the experiment, in: a- flux, b- concentration of species.

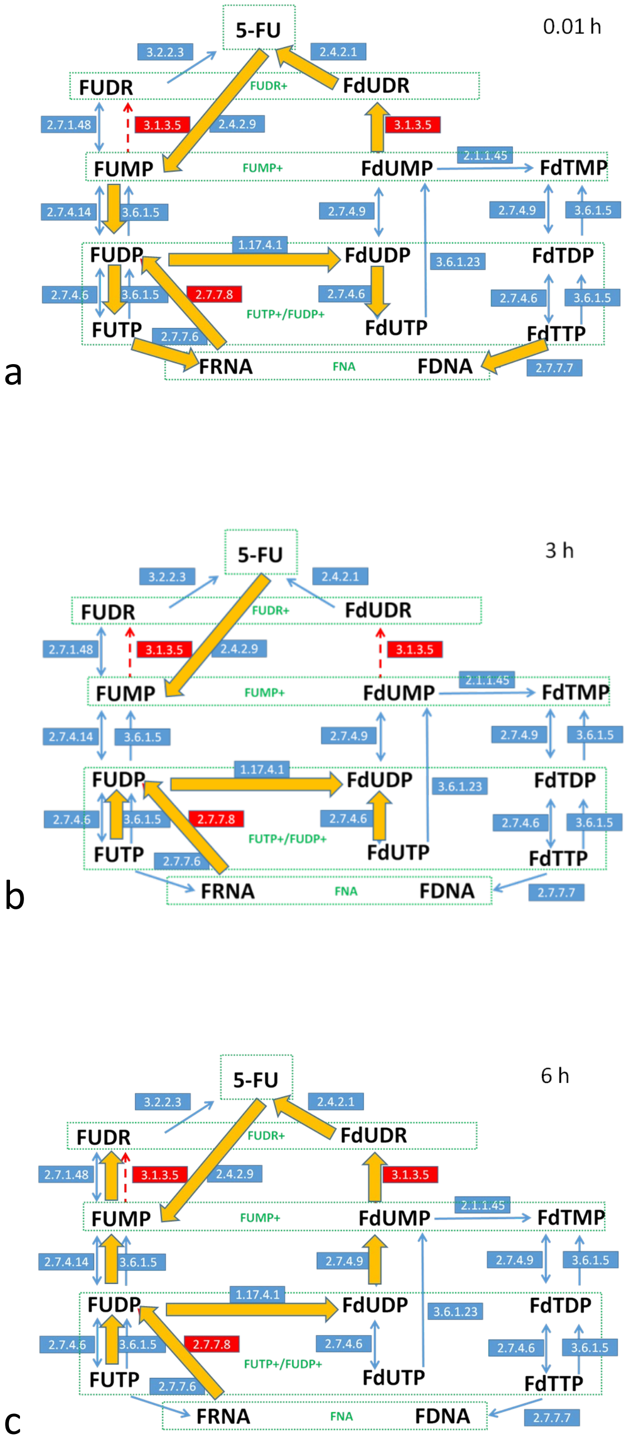
0.01 h
5-FU
3.2.2.3
2.4.2.1
FUDR
FUDR+
FdUDR
2.7.1.48
3.1.3.5
2.4.2.9
3.1.3.5
2.1.1.45
FUMP
FUMP+
FdUMP
FdTMP
2.7.4.14
3.6.1.5
2.7.4.9
2.7.4.9
3.6.1.5
1.17.4.1
FUDP
FdUDP
3.6.1.23
FdTDP
2.7.4.6
3.6.1.5
2.7.7.8
2.7.4.6
2.7.4.6
3.6.1.5
FUTP+/FUDP+
FUTP
2.7.7.6
FdUTP
FdTTP
FRNA
FNA
FDNA
2.7.7.7
a
3 h
5-FU
3.2.2.3
2.4.2.1
FUDR
FUDR+
FdUDR
2.7.1.48
3.1.3.5
2.4.2.9
3.1.3.5
2.1.1.45
FUMP
FUMP+
FdUMP
FdTMP
2.7.4.14
3.6.1.5
2.7.4.9
2.7.4.9
3.6.1.5
1.17.4.1
FUDP
FdUDP
3.6.1.23
FdTDP
2.7.4.6
3.6.1.5
2.7.7.8
2.7.4.6
2.7.4.6
3.6.1.5
FUTP+/FUDP+
FUTP
2.7.7.6
FdUTP
FdTTP
FRNA
FNA
FDNA
2.7.7.7
b
6 h
5-FU
3.2.2.3
2.4.2.1
FUDR
FUDR+
FdUDR
2.7.1.48
3.1.3.5
2.4.2.9
3.1.3.5
2.1.1.45
FUMP
FUMP+
FdUMP
FdTMP
2.7.4.14
3.6.1.5
2.7.4.9
2.7.4.9
3.6.1.5
1.17.4.1
FUDP
FdUDP
3.6.1.23
FdTDP
2.7.4.6
3.6.1.5
2.7.7.8
2.7.4.6
2.7.4.6
3.6.1.5
FUTP+/FUDP+
FUTP
2.7.7.6
FdUTP
FdTTP
FRNA
FNA
FDNA
2.7.7.7
c

9 h

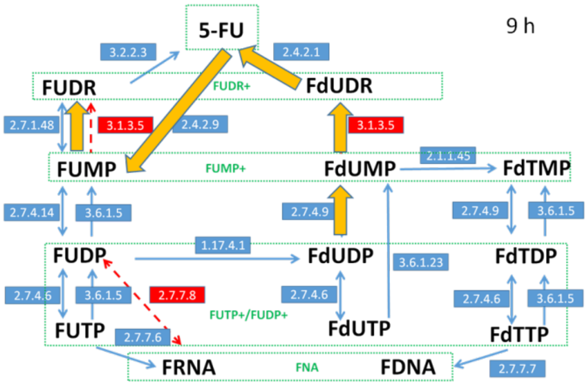

d

Supp. Fig.4.The main metabolic routes during experiment. The orange arrows indicate the dominating flux predicted by the fitted model, for the different time of the experiment: a - 0.01h, b- 3 h, c- 6 h, and d- 9 h.

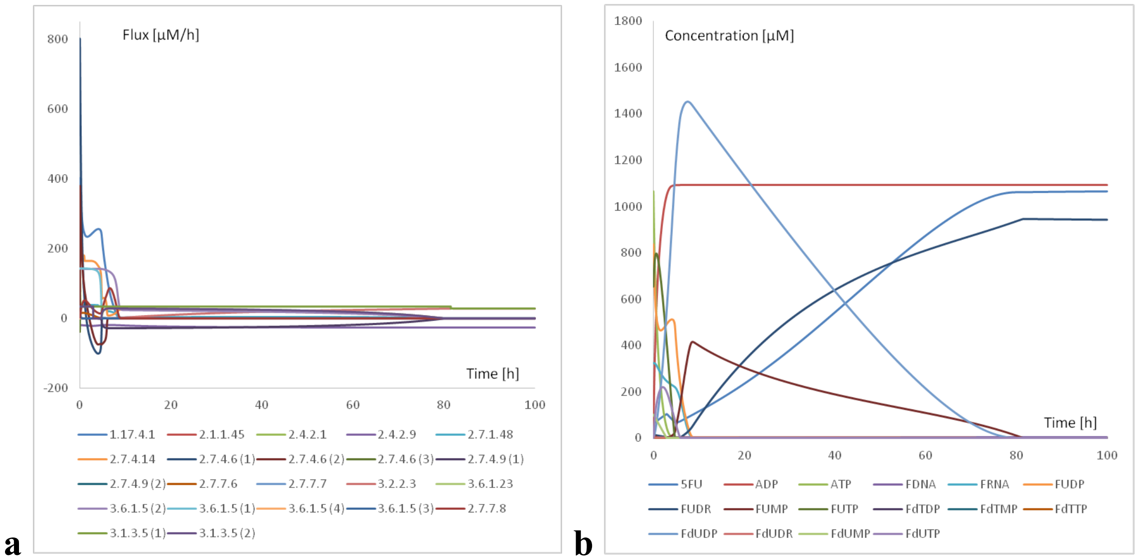

Supp. Fig.5. Theoretical predictions of fitted model (9h< time <=100h). Kinetics of changes, in: a- flux, b- concentration of species.

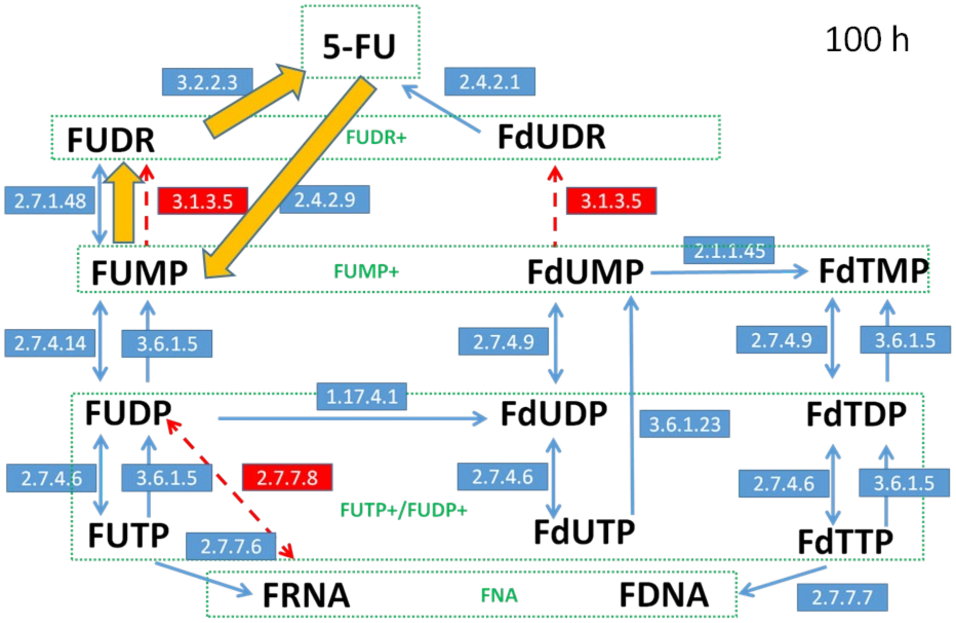

Supp. Fig.6.The main metabolic routes beyond the experiment. The orange arrows indicate the dominating flux predicted by the fitted model, time = 100 h.

Supp. Tab.1. Metabolite initial concentration [µM]					
**No.**	**Name**	**Bfv**	**Avg**	**Stds**	**Dev/Stds**
1.	5FU	5.5623E+01	5.5613E+01	1.3E-02	0.78
2.	ADP	2.789E+01	2.829E+01	3.9E-01	1.05
3.	ATP	1.065370E+03	1.065354E+03	2.4E-02	0.69
4.	FUDR	1.3868E+01	1.3890E+01	1.2E-02	1.02
5.	FdUDR	8.504E+00	8.505E+00	1.2E-02	0.11
6.	FUMP	1.652E+01	1.664E+01	1.1E-01	1.13
7.	FdUMP	9.9743E+01	9.9730E+01	1.9E-02	0.70
8.	FdTMP	1.370E-01	1.399E-01	4.2E-03	0.69
9.	FUDP	8.38078E+02	8.38025E+02	8.0E-02	0.67
10.	FdUDP	8E-10*	2.8E-06	2.9E-06	0.99
11.	FdTDP	1E-11*	6.7E-09	4.5E-09	1.51
12.	FUTP	6.54891E+02	6.54869E+02	3.1E-02	0.71
13.	FdUTP	5.6E-05	4.9E-05	1.2E-05	0.62
14.	FdTTP	1.758E+00	1.743E+00	1.9E-02	0.83
15.	FDNA	1E-05*	1.2E-03	1.3E-03	0.96
16.	FRNA	3.21859E+02	3.21848E+02	2.9E-02	0.38

* means no statistical significance
